# Editorial Department

**Published:** 1881-10

**Authors:** 


### The Bistoury.

ELMIRA, N. Y., OCTOBER, 1881.

The BISTOURY is published Quarterly, upon the first of April, July, October and January, at Fifty Cents a year in advance.

### Editorial Department.

A NATION MOURNS.—Throughout the land the news, carried by electric wings, spread ere midnight, and the solemn tolling of the bells awakened many to grief for the man and anxiety for the nation.

Hope, faith and prayer had sustained the people through the fitful phases of the President's illness; the necessity of this one life seemed to warrant almost the expression, "he *must* not, he *shall* not die." But human wishes and human efforts fail before the inevitable, and this day (September 20th,) the people bow in woe, and look beyond themselves for the future of our country, whilst they drape their houses with crape and mourn in the depths of their hearts for the loved one dead.

Called to the highest post in our Republic, at a time when our institutions were in peril from the struggles for power of political giants who would rule or ruin; he assumed the reins of government and quietly but surely adopted a policy which won for him the confidence of the people and the opposition of those whose strength was in the continuance of the bad into which our political affairs had gradually drifted. Under his rule the people, without regard to party lines or sectional differences had begun to develop a prosperity which seemed unbounded, and our country's future was foreshadowed in a brilliancy brighter because of the corrections of errors that had dimmed its past.

The people's love went out to this man—it seemed as if nothing was wanting to complete his greatness—he had reached his zenith. No, the assassin's bullet carried him a step higher in his career here, and sent him loaded with worldly honors and human love to his reward above.

His history is before us, replete with inci dent and full of example to young men starting out in life.

His early career was marked by successful struggles with disadvantages which surrounded him. Success in one, gave capital and energy to overcome the next—with constant self reliance and steady adherence to right, he pushed forward, gathering friends and power until he reached not only the highest political honors of the Nation, but still greater was his reward, he died without a rival in the hearts of his people. His name will ever be associated with those who gave their lives for their country and stands recorded among the heroes of the Nation.

The few months of his administration marked him as a man among men, giving pride to his supporters, and rallying around him the best elements of all parties. Quietly, without any meetings, any formulated ceremonies, signs or passwords, the better men of this Republic, almost unknown to themselves are now arrayed in one determined brotherhood to weed out all that disgraces our political status, and from the life and death of James A. Garfield may spring a lasting prosperity, which we shall owe to the awakening of the people by this great sacrifice.

Ought we to mourn for him ? Can such a fate suggest sorrow for the individual ? For the loved ones of his family—*our* family now, for it belongs to the nation—for ourselves and our country, we must mourn the loss of such a man, but for him, death has come at a time to make his name immortal.

To those who succeed him—to the one man standing alone just now in the succession, the people look with hope and fear. His position is a trying one, but shaking off all trammels that may have heretofore surrounded him, calmly contemplating his position as chief ruler of the greatest nation on the earth ; assuming his power with a knowledge of the fearful responsibilities thereto attached, with the help of Almighty God, which has carried our country to the eminence among nations, which it has already reached, may we not hope that it will be given to him to do right ? That he will see the will of the people and the prosperity of the country in the continuance of the policy of his predecessor, and find himself at no distant day in a position when the people will heartily repeat the expression of our neighbors, "The King is dead, long live the King!"

THE NINETEENTH OF THE MONTH, seems to have been an eventful day for President Garfield. He was born on November 19th, 1831, was made a Major-General on the 19th of September 1863, and died September 19th, 1881.

---

THE NORTH AMERICAN REVIEW for October is before us. It contains eight excellent, entertaining and instructive papers by the very best talent our country affords. The Review is, par excelence, *the* literary journal of this country.

---

MIS-NAMED.—Some one advertisers in one of our city newspapers for "sales-ladies." This reminds us of the little vagrant who applied to us recently for alms, and of whom we inquired her mother's occupation, the answer was, "she's a wash-lady."

---

DR. BLISS AND DR. HAMMOND.—Dr. Bliss is reported as saying that Dr. Hammond's treatment of the former was "scandalous and ungentlemanly." We wonder what sort of adjectives would apply to the conduct of Dr. Bliss toward Dr. Baxter ?

---

COOLING A WATERMELON.—A friend recently invited acquaintances to partake of a particularly fine watermelon. In order to cool it the more thoroughly, he placed it under the water faucet in the kitchen, permitting the water to flow upon it until his guests should arrive. Imagine his discomfiture when, at the expiration of a couple of hours, he discovered he had turned the hot water cock upon the melon ! Boiled watermelon was unanimously voted a complete and utter failure !

---

PROBING FOR BULLETS.—Dr. Hammond is reported as saying:—"I never had any hope of his recovery since the 23rd of July, and I had little hope after the first forty-eight hours, because the bullet was not located and an operation made to extract it while the President was strong."

Indeed ? Suppose the surgeons had been fools enough to have followed your advice. What good could have come of the probing and cutting for a bullet, the location of which was widely apart from where it would have been searched for ? Then, since the bullet was encysted, and therefore harmless, what good would have resulted from such meddlesome interference, anyhow ?

THAT "INDUCTION BALANCE."—The autopsy in the President's case, has afforded a series of surprises, the most glaring of which was the stupendous inaccuracy of that induction balance which was supposed to have located definitely the situation of the bullet in the President's body. Could there have been anything more absurd ? Blunders like these shatter the confidence of the people in the reliability of the opinions and devices of so-called scientific men.

---

THE CONTRIBUTIONS.—Our contributors—all of them—have most excellent articles in this number of the BISTOURY. Do not skip one of them. We doubt whether any magazine comes to your hands containing as much interesting and valuable reading as is afforded in this journal. A new contributor appears in this number, who, from the vigorous manner in which he handles his subject in his opening article, we judge he is likely to prove sprightly and entertaining.

---

A BEAUTIFUL SIMILE.—Of the many expressions of the late President that are being printed, none are more poetical or beautiful of couception than this :

"Individuals may wear for a time the glory of our institutions, but they carry it not to the grave with them. Like raindrops from heaven, they may pass through the circle of the shining bow and add to its luster, but when they have sunk in the earth again, the arch still spans the sky and shines gloriously on."

---

THE BISHOP AND THE SQUIRREL.—C. tells us the following good story which should be placed on record. Years ago, when he was a boy, he was the happy possessor of a pet squirrel. The little animal was very tame and had the freedom of the house. Upon one occasion, a catholic Bishop, wearing the old time farmer's satin pants—loose and flowing—[there being room in each one of the pantaloon legs for both of the Bishop's,] called upon certain lady members of the household, who occupied apartments upon the second floor of the dwelling. He was earnestly and pleasantly engaged in conversation with the ladies, when the pet squirrel came into the room unobserved, and, finding the Bishop's pant legs wide and roomy, sought to explore them to the bifurcation, which he accomplished without delay, to the consternation and great fright of that amiable gentlemen. He quickly grasped the animal through the surrounding material and called upon one of the ladies to reach for and dislodge it from its hiding place. But, the more the lady pulled the squirrel's tail, the tighter he fastened his claws in the affrighted Bishop's leg. At last, the animal, after much coaxing and more pulling, was dislodged, but not without great embarrassment to the ladies and their clerical caller. C., when he found his pet's tail bare as a liberty pole—every particle of the hair and skin having been stripped from it, demanded an explanation of the causes leading thereto, and obtained the recital of the above particulars.

---

A SILVER MEDAL.—There exists in this city a society of gentlemen who style themselves the "Old Sledge Club." There are just eight of them, and are sufficiently advanced in years to permit one of their number to indulge in a silver wedding. The remaining seven presented the lady with a handsome silver service, while the groom was the recipient of a medal answering to the following description: On one side a heart pierced with an arrow, over which the word "Lost," and below, "In 1856." On the reverse side, the jack of clubs, over which the word "Lost," and below, "In 1881." Since the member celebrating his anniversary was perpetually "losing his jack," he was the better able to appreciate this delicate reminder of the circumstance.

---

EMPHATICALLY SETTLED.—In taking a sleeping coach the other night, a party entered the train who held a ticket for a berth already occupied by a sleepy passenger.

"Halloo here!" exclaimed the new comer, shaking the sleeper roughly to arouse him to a sense of the situation. "Halloo here, old fellow, this is *my* berth!"

"Not much," said the aroused individual, as he poised his body on one hand and rubbed his eyes with the other;—"Not much, neighbor, I engaged the berth, got a ticket for it, paid for it, and by thunder I'm *in* it!" He then drew the blankets more closely about him, turned his back on the intruder, ducked his head into the pillow and remained master of the situation.

---

THE AMERICAN SOCIETY OF MICROSCOPISTS, which met in Columbus Ohio, last August, held a very delightful meeting, elected Dr. Geo. E. Blackham of Dunkirk, N. Y., President, and selected Elmira as their place of meeting for next year.

Dr. Blackham is already exerting himself to make the next meeting of the Society a notable one—rich in papers and large in attendance of members, while the Elmira Microscopical Society are taking measures for suitably entertaining the National Society. The meeting of next year will doubtless be the largest and most interesting of any yet held. The Court House, Elmira College and lecture room of Park Church have been offered for the meetings, while the Elmira Society have taken measures to entertain all the delegates and to give them a suitable "reception" during their stay in this city.

---

SECOND SIGHT.—Dr. S. A. Sterrett relates a case of so-called second sight, in the Pittsburg Medical Journal. The patient was an old man, 83 years of age, who had been blind from cataract, was operated upon and restored to vision, when he could read Jæger's No. 3, test types by aid of $+2\frac{1}{2}$ spectacles. The Doctor then proceeds as follows:

"He continued to use glasses for eight years, when, singular to relate, he threw aside these aids to vision, and began to use his unaided eyes. For the last two years of his life, for he died recently at the age of 93, he could see to read the finest print without glasses of any kind. He had a perfect restoration of sight at the age of 91, and that in eyes devoid of crystalline lenses.

The theory that second sight is caused by some peculiar change in the lens of some old people—that the lens by inhibition of the surrounding fluids become more convex and thus attains a higher degree of refracting power—does not seem to answer in this case, as the lenses had been removed ten years before.

By what theory then can we explain the phenomena of second sight as exhibited in this case?"

The explanation is easy. Had the Doctor examined his patient, he would have found that the pupils had contracted to a very minute diameter, thus excluding the more divergent rays of light, so that the cornea, and the large double convex lens formed by the concave posterior surface of the cornea and the concave anterior surface of the vitreous, were sufficient to refract the more parallel rays and focus them at the proper point on the retina.

Either a very small iridectomy was performed in this case—concealed under the upper lids—or the lenses were depressed and not extracted, rendering a mutilation of the pupil unnecessary.

---

GUITEAU.—An article is being copied into the papers purporting to emanate from a brother-in-law of the assassin, arraigning Col. Corkhill, the District Attorney, for expressions made use of by him with regard to his knowledge of an organization for the prompt punishment of Guiteau, should he be cleared upon the quibble of insanity. Without referring to the bad taste of the writer in his public acknowledgement of his unfortunate relationship, or the questionable propriety of newspapers being made vehicles for appeals in favor of such a criminal, we would like to call attention to something beyond the mere legal definition which this member of the Chicago bar gives to the word "punishment."

He says:—"Punishment is the penalty for crime, and crime cannot exist without that malicious intent which is bred in a sound mind." Then follows the claim for a fair trial and acquital, if a jury agree to give a verdict of insanity.

The Court Annals have plenty of cases of crimes committed, insanity affected and proven legally, prisoners sent to insane asylums; a convenient period served there, restitution to health (?) and liberty regained.

Now, what is "punishment" for ; why is the death penalty inflicted ? certainly not as a matter of public vengeance, clearly not because the officers of the law desire the job or the majority of the people wish to witness it.

In addition to putting out of the way, from doing further harm, a murderous villain, "punishment" is a warning to others, arresting them on the downward path which Guiteau followed until he became a murderer.—"*Salus populi suprema est lex*"—we are all in the habit, and justly too, of saying, when a person outrages public sentiment by some gross violation of others rights, "he must have been out of his mind." It is not improbable that insanity in some stage or other may be the cause of most crimes ; but, must all crime go unpunished on that account ? Is the Nation to be struck to the heart with impunity, because rum, fanaticism and general depravity have prepared a scoundrel for the damnable work of an assassin ?

Are we to read sentimental pleadings for a fair trial, and attacks in advance upon the prosecuting attorney in the case of a villain like Guiteau, of whose guilt there is no doubt, and whose crime ought to be considered ten thousand times greater than if his pistol had been discharged at a private individual ?

It is a subject of regret that summary justice had not overtaken him on the spot, but if the law now *must* be vindicated by a formal trial, at least, let not the patience of the people be tried by appeals in his favor from relatives, who should be glad to be rid of the brute.

Many noble hearted fellows, honest and true, went out to certain death in forlorn hopes, for the sake of their country, shall we plead for the life of this assassin whose victim was our country's hope ?

---

OUR SEWERAGE—A sanatory engineer has just completed a survey of this city, and has recommended, in an elaborate report to the Common Council, a system of sewerage that shall conduct all the sewage of the city to a common outlet that will discharge itself into the river, jnst within the city limits.

Now, there cannot be the slightest doubt that Elmira is badly drained, and that what sewerage we have is absolutely worse than none, since it empties into the river within the city, distributing its filth and malarial influences along one of our most populous thoroughfares. But, the scheme to expend sixty thousand dollars for a system of sewerage that will collect and conduct a largely increased and dangerous filth only a little further down the river, to poison our farming population below, kill the fish, sicken the cattle, and endanger the people in villages along our water course, becomes a questionable improvement. Better than this, and infinitely less costly, would it be to continue and enlarge upon our old system of cess-pools, particularly since the introduction of water works has enabled us to abandon as a means of water supply, our already dangerous wells. Our city being underlaid by a stratum of gravel, permits of a perfect and safe means of drainage, and one that need not subject the city to any expense save in such buildings as are owned by the people in common.

A cesspool, sufficiently large, sunken well into the gravel, securely walled and covered, should and would afford drainage for the kitchen and closets of any dwelling in our city for the lifetime of its occupants. We can cite numerous instances where such structures have performed their duty effectually and wholesomely for a long series of years. In view of these facts, the system of sewers proposed by the engineer referred to, seems to be an extravagantly costly undertaking, and one that will by no means increase the healthfulness of our city as an abiding place.

It is a marvel that, in this intelligent age, the city authorities should have permitted a large state institution to empty its filth, from a large sewer, directly into the most populous portion of the city ! That this sewage should be conducted, without delay, outside the city limits, seems plain ; but that other and larger sewers should be constructed to increase the mass of filth to be deposited in the river, needs to be carefully considered, and if possible, prevented.

---

THE ATTENDING SURGEONS.—Now, that the President is dead, there will be no end to criticism and perhaps condemnation that will be heaped upon the professional gentlemen who conducted his case. It is not wholly harmless amusement, nor is it generous to croak about the treatment and make suggestions of other methods that *might* have resulted differently. No matter what Dr. Hammond or any other discourteous meddler may say about probing for and cutting out the offending ball—it was good surgery to let it alone, particularly, since it was not definitely known just where it was lodged. Experienced surgeons know and can testify to the fact that more harm has been done in probing for bullets than the missles have done when left undisturbed. [Since penning this sentence, the results of the autopsy have been made known, revealing the bullet completely encysted—so causing no harm to the patient but exhibiting the evidence of Dr. Hammond's error in recommending its removal.] We were one of the few who confidently believed in the illustrious patient's final recovery, and but for the lung complication which made embolism possible, we do not believe even now, that he reached that degree of prostration consequent upon blood poisoning, but that recovery was possible had not the heart's failure occurred. [Here again, the facts revealed show that the cause of death was secondary hemorrhage, a circumstance that does not change our conclusions.]

Let us be generous toward the surgeons. They did their best. They acted wisely and courageously in the face of much discouragement. They are able men—no better surgeons can be named. They feel the loss even more keenly than do we. Let us bury our dead—mourn deeply our loss, and discharge our surgeons with our grateful thanks for their earnest and skillful endeavor.

---

NOT AN EVIL.—It too frequently occurs that by our zealousness in some particular belief or theory, persons cultivate within themselves a belief that because others differ with them entirely on important subjects, such a difference proves not only the error of thought in the individual, but also constitutes him a bane to society and one from whom nothing but evil can result.

Our attention was called to this by a conversation in which the name of Robert Ingersoll was mentioned, and a prompt disapproval of the man and his lectures was announced by one present whose mind, as manifested by his remarks on other subjects, seemed to take a very liberal view of worldly affairs.

The other side of the question was taken by another who did justice to Mr. Ingersoll's great talents as a speaker, and made a claim for him which possibly he would not make for himself, but which we give our readers as a subject for discussion amongst themselves.

The assertion was that "Robert Ingersoll, instead of doing harm was in reality doing good, that with all his wonderful ability in argument and oratory, he never could make the least impression against the truth, against pure religion—these were invulnerable—but that the result of his efforts would eventually be beneficial to mankind."

Now the speaker did not claim for Mr. Ingersoll that he was an apostle of truth, self appointed to do good. Nor, let us say, did he assert the opposite—but his argument was that God seemed to permit what appeared blasphemous and evil to many to exist, and out of that evil He wrought good.

The ways of Providence are inscrutable, and we cannot judge them from our limited human comprehension. Many things daily occur to those who seem to walk upright before God and man, which appear to warrant cavil at justice from the Source whence we all hope for justice, and frequently time develops the mercy which governed the blow, sometimes we never recognize the mercy but nevertheless it exists.

Suppose in an early future, men of thought should be able to worship Almighty God, not only in conceded faith, but in a faith going hand in hand with mind, with the powers of reasoning given them to judge on all other subjects; reason untrammelled by questions which men like Ingersoll have the power to answer with ridicule, and unfortunately oftimes with truth.

If such a result should be brought about by Mr. Ingersoll's oratory—if men on religious subjects shall have false religion so weeded out that they need not be afraid to think, then certainly he will have done good.

We are not praising Mr. Ingersoll, nor are we condemning him—merely accounting for his permission to exist, if really he be dammed-worthy, as many pious souls are want to believe. He may exhaust his brain in argument, and fill his speeches with beautiful rhetoric, but his triumphs must always be over evil; never can he overthrow truth; *Magna est Veritas et prævalebit.*

---

WHISTLING.—There be those who whistle for fun—from an exuberence of unrestrained joy. Others whistle thoughtfully, in a low, measured tone, as with hanging heads, they journey moodily toward home from the perplexing business of the day. Boys whistle just to stir up the folks—to let them know they are on hand. Girls whistle in a coquettish, musical sort of tone to attract the passing beaux. Everybody whistles at times, for some reason, or other, when there is method if not music in the endeavor, but a locomotive engineer causes his machine to whistle out of sheer cussedness.

These reflections are induced through long suffering from the whistling nuisance. The denizens of the 5th Ward in this city, are most patient sufferers under the most diabolical of inflictions from locomotive whistles. The city is wonderfully careless and remiss in its duty toward these long suffering people. A short distance below the Railroad bridge is a junction where two roads diverge from the Erie road-bed, to which point signals must be conveyed to notify the switchman, on duty there, to open the switch. These signals are given through an unearthly and ear-piercing system of whistlings, commencing on the bridge and continued to Hudson street, and below. No one can have any conception of this distressing noise, until they have tried to sleep, or sit up with a sick person, anywhere within the jurisdiction of this whistling abomination. We have known of numerous cases of great injury having been caused to sick people by it. We have had ample cause to damn it ourselves and to aid others in the same praiseworthy effort. It should be stopped. There is no necessity for it. The signals can be noiselessly conveyed by telegraphic or telephonic means. We are sure that the gentlemen conducting the Railways in this city need only to have their attention called to this screaming evil and the absolute distress it inflicts upon our people, sick and well, to have it modified or wholly eradicated.

---

THE STATE FAIR.—Once in three years, the State Agricultural Society holds an exhibition in this city. The county expended a large sum of money to provide said society with permanent exhibition grounds. The action of the county commissioners, has been unfavorably criticised in this matter, many tax-payers claiming that these meetings are of doubtful utility to the city or county. Perhaps the State Fair, as it is usually named, does not benefit our merchants, pecuniarily, as much as was anticipated; nevertheless, the fair, with its thousands of visitors, curious contrivances, herds of fine cattle, stables of well-bred horses and pens of sheep, swine and poultry, affords an exhibition that must be of great advantage to our farming community, stimulating them to better endeavors in their chosen pursuits. It presents an innocent sort of amusement too, where the people of all shades of intelligence and complexion may congregate to chat upon all themes near or remotely allied to agricultural matters. It makes a convenient pic-nic ground, where the country girls climb into the brand new wagons, poke their dainty feet through the enameled dashers while they munch their luncheons, and shy their cheese rinds at the idle loiterers about them. Even the little pigs, as they partake of the natural nourishment provided for them, seem to enjoy their surroundings the more keenly, while mother pig grunts approvingly and proudly over the proceeding. The well groomed bulls, and the sleek, and mild eyed cows show off their best points to the admiring crowd, and the little calves blate and bawl until a share of admiration and suitable caressings are bestowed upon them. The chickens, spangled, striped, hooded and plain, cackle cheerily within their coops, and scratch sawdust into the eyes of the passers by. Roosters, big and little—stalwarts and half-breed—vie with each other in defiant calls, and the impudent quack! quack! of the slovenly duck causes a smile of derision from the medical inspector. And so, the great fair affords a healthful play day for man and beast, in consideration of which, it is not without its advantages—not wholly useless.

PAY UP.—You should be ashamed to be in arrears with your subscription to the BISTOURY when so small a sum is asked for it. If you cannot send 50 cents conveniently, enclose a dollar, and be credited for two years.

DR. GEO. E. BLACKHAM—the President elect of the American Society of Microscopists is a most opportune and happy selection for that Society. Di. Blackham is in every way fitted for the position. No one in this country has a better knowledge of the Microscope, nor do there exist any more skillful manipulators than he. His paper upon "Angular Aperature of Microscopic Objectives," read before the Microscopical Congress at Indianapolis, and since published in book form, has given him a world wide reputation as a scientific Microscopist. The Royal Microscopical Society, of England, recognizing his eminent ability, have made him an honorary member, so that the Doctor ornaments his name with a "F. R. M. S."

The Doctor is a young man—not yet thirty—and has the necessary vim and executive ability to make the National Society a vigorous and healthy one. He is constantly issuing circulars and writing letters to the members of the Society, stirring them up and urging them to prepare papers for the next meeting. We may be sure that so long as he remains at the head, the American Society of Microscopists will be a pronounced and complete success.

GAMBLING.—This propensity seems almost inherent in our nature. Games in which chance forms the attractive part are indulged in with avidity in early childhood, and chance insures constant votaries in after life, whether it comes in the game of "snatch bags" at church fairs, lotteries, the gaming table or the exchange. We quote the following statement from another journal:

"In no part of the world probably, has the science of Sociology been studied so closely and with so much advantage as in France. For more than two centuries the policies of prohibition and licensing have been alternated in Paris. Infamous penalties were attached to gambling under Louis XI, when it was strictly prohibited. * * * Under the Republic it was again prohibited, and in 1806 Napoleon turned the whole matter over to the police to be regulated as they might find best. Under the restoration the city of Paris agreed to pay the Government 5,500,000 francs, (about $1,100,-000) a year, for the privilege of "farming" out the gambling of the Capitol.

The leases expired in 1837, and the whole matter having been debated at length in the Chamber of Deputies, it was finally resolved that the gambling houses of the Capitol should be closed on 31st December, 1837.

The Finance Minister in consenting to this motion warned the Deputies that it would be idle to expect the suppression of gambling from the measure. "Close Frascati's" he said, "and you will find the better classes hurry to gamble at the Exchange where fortunes will be lost and won more easily, more frequently, and no whit more morally." The Baden-Baden an Hamburg of to-day, the Frascati's and Crockfords of the past are but trivial sources and centres of moral corruption in comparison with the Stockboards of the Old and New World."

This article seems particularly fitted for quotation at this time, when speculation runs higher than ever before, not only at the stock boards, and grain exchanges, but in every article entering into general use or likely to be in demand. We heard of a corner in crape in one of our large cities before the President's death.

Perhaps we may sometime arrive at the conclusion that vices that cannot be eradicated, had better be controlled by license and law.

WORTHY OF RECORD—The Powell Manufacturiug Co., of Baltimore, the manufacturers of POWELL'S BEEF, COD LIVER OIL AND PEPSIN, the superior food and nutritive tonic, have taken the initiative in the introduction of their valuable medicine, (which our leaning practitioners are prescribing largely), by guaranteeing to the medical profession that they will not in any way advertise the POWELL'S BEEF, COD LIVER OIL AND PEPSIN so that it will come under the head of a patent medicine—*Exchange.*

